# Loss of Group II Metabotropic Glutamate Receptor Signaling Exacerbates Hypertension in Spontaneously Hypertensive Rats

**DOI:** 10.3390/life11070720

**Published:** 2021-07-20

**Authors:** Julia Chu-Ning Hsu, Shinichi Sekizawa, Ryota Tochinai, Masayoshi Kuwahara

**Affiliations:** Department of Veterinary Pathophysiology and Animal Health, Graduate School of Agricultural and Sciences, The University of Tokyo, Tokyo 113-8657, Japan; juliahsujh@smail.nchu.edu.tw (J.C.-N.H.); ar-tochinai@g.ecc.u-tokyo.ac.jp (R.T.)

**Keywords:** autonomic nervous function, baroreflex, group II metabotropic glutamate receptors, heart rate variability, nucleus tractus solitarius, blood catecholamine, ultra-sonography

## Abstract

High blood pressure is a major risk factor of cerebro-cardiovascular outcomes. Blood pressure is partly regulated by the autonomic nervous system and its reflex functions; therefore, we hypothesized that pharmacological intervention in the brainstem that can regulate blood pressure could be a novel therapeutic strategy to control hypertension. We infused a group II metabotropic glutamate receptor (mGluR) antagonist (LY341495, 0.40 μg/day), using a mini-osmotic pump, into the dorsal medulla oblongata in young spontaneously hypertensive rats (SHRs), as this area is adjacent to the nucleus tractus solitarius (NTS), of which the neurons are involved in baroreflex pathways with glutamatergic transmission. Blood pressure was recorded for conscious rats with the tail cuff method. A 6-week antagonist treatment from 6 to 12 weeks of age slightly but significantly increased systolic blood pressure by >30 mmHg, compared to that in SHRs without treatment. Moreover, the effect continued even 3 weeks after the treatment ended, and concurred with an increase in blood catecholamine concentration. However, heart rate variability analysis revealed that LY341495 treatment had little effect on autonomic activity. Meanwhile, mRNA expression level of mGluR subtype 2, but not subtype 3 in the brainstem was significantly enhanced by the antagonist treatment in SHRs, possibly compensating the lack of mGluR signaling. In conclusion, mGluR2 signaling in the dorsal brainstem is crucial for preventing the worsening of hypertension over a relatively long period in SHRs, through a mechanism of catecholamine secretion. This may be a specific drug target for hypertension therapy.

## 1. Introduction

Hypertension is one of the major risk factors of cerebral and cardiovascular outcomes. Various interventions, such as diet control and exercise, have been recommended to patients with hypertension as well as healthy subjects to prevent progression of blood pressure abnormalities [1]. Although there are many drug treatment options for hypertension, half of the patients treated have not reached target blood pressure levels, suggesting the involvement of unknown mechanisms which have not been targeted by existing drugs [2].

Hypertension can result from various factors, such as blood fluid volume, cardiac output, blood vessel stiffness and arterial smooth muscle tones [2]. Among them, vascular tone is mainly regulated by the sympathetic nervous system, but is also reflexogenically controlled by afferent information from baroreceptors. This afferent information is transmitted to neurons in the NTS, caudal ventrolateral medulla (CVLM), rostral ventrolateral medulla (RVLM) and then to the intermediolateral nucleus [3]. In fact, RVLM, which is the source of sympathetic outflow, can be an antihypertensive target of clonidine, an alpha-2 adrenergic receptor agonist [4]. Therefore, managing sympathetic nervous activity might be key to maintaining blood pressure within a normal range.

The baroreflex mechanism regulates heart rate (HR) and blood pressure to maintain the whole-body system functioning [5,6]. As baroreflex sensitivity is found to be reduced or impaired in hypertensive subjects [7,8], a loss of physiological mechanisms may be involved in baroreflex signaling pathways. In the baroreflex pathways, synaptic transmissions between neurons are either glutamatergic or GABAergic [9]. However, it is considered that NTS neurons are mainly projecting glutamatergic fibers to neurons in the CVLM or other nuclei [10]. At glutamatergic synapses in the NTS, glutamate is the main neurotransmitter, and the receptors are ionotropic (i.e., AMPA and NMDA receptors), while G-protein-coupled metabotropic glutamate receptors (mGluRs) are expressed within or near synapses to modulate synaptic transmission and neuronal excitation of relatively longer durations [11,12]. mGluRs are traditionally divided into three groups, based on structure and physiological function [13]. Interestingly, group II mGluRs (mGluR subtype 2 and 3: mGluR2/3) are both presynaptically and postsynaptically expressed, and function in the NTS [14,15], suggesting that these receptors may be greatly contributing to neuronal signaling. Microinjection of mGluR modulators into the rat NTS could dynamically change blood pressure; however, there are several contradictions between cardiovascular response and mGluR agonistic/antagonistic effects [16,17,18]. 

As described above, hypertensive patients show impaired baroreflex function, which could only be related to transient regulation; thus, chronic modulation/modification of blood pressure regulatory mechanisms might be responsible for the development of hypertension. As neural mechanisms of blood pressure regulation are largely controlled by glutamatergic transmission, including the baroreflex mechanism, we hypothesized that group II mGluRs in the dorsal medulla oblongata, including NTS, could be a key factor to chronically regulate blood pressure, especially during hypertension development. To test this hypothesis, we used young spontaneously hypertensive rats (SHRs), which have been widely used in hypertension studies [19]. The reason that we used young SHRs was that they start to develop hypertension at approximately 6 weeks of age, up until the following >6 weeks [20,21]. 

For mGluRs intervention, we chronically applied a selective mGluR2/3 antagonist, LY341495, into the dorsal medulla oblongata with the aid of an osmotic pump to block endogenous glutamate in rats during 6–12 weeks of age. In our previous study, we found that mGluR2/3 agonist treatment into the dorsal medulla oblongata could suppress the development of hypertension [22]. However, as NTS microinjection of the mGluR2/3 antagonist could also decrease mean blood pressure by approximately 18 mmHg in normotensive rats [18], the current study was considered very important for understanding pathophysiological mechanisms of blood pressure regulation. BP and HR were non-invasively measured by the tail-cuff method throughout the developmental stages of hypertension. After the antagonist treatment, autonomic nervous activity was assessed by the power spectral analysis of heart rate variability (HRV), using a radio-telemetry system. Cardiac and renal functions were evaluated with ultrasonography and blood catecholamine level, and messenger RNA expression of mGluRs2/3 in the brainstem was also evaluated.

## 2. Materials and Methods

### 2.1. Animals

All experimental protocols were approved by the animal care and use committee of the University of Tokyo (No. P17-033). Animals were used according to the Guidelines for the Care and Use of Laboratory Animals established by the Graduate School of Agriculture and Life Sciences at the University of Tokyo. Four-week-old male SHRs (total 38 animals) and Wistar Kyoto rats (WKYs) (total 15 animals) were purchased from Charles River Laboratories Japan, Inc. (Yokohama, Japan), and kept in a temperature-controlled room (24 °C ± 1 °C) under automatically controlled lighting (light on: 0800–2000 h) with free access to food and water.

### 2.2. Dorsal Hindbrain mGluR2/3 Treatment

All surgical procedures were performed under a specific level of isoflurane anesthesia (Pfizer Japan Inc., Tokyo, Japan), i.e., the loss of withdrawal reflexes. Six-week-old animals were implanted with a mini-osmotic pump (ALZET Model 2006, DURECT Corporation, Cupertino, CA, USA) that can continuously deliver contents for 6 weeks. The pump was removed at the age of 12 weeks. For the implantation, the lateral cervical space was opened, and a catheter (external diameter 0.61 mm, internal diameter 0.28 mm) connected to the mini-osmotic pump was inserted in the cranial cavity through the foramen magnum. The tip was located near the caudal end of the medulla oblongata. The pump was set under the skin of the back, which was filled with LY341495 ((2S)-2-amino-2-[(1S,2S)-2-carboxycycloprop-1-yl]-3-(xanth-9-yl) propanoic acid, Tocris Bioscience, Bristol, UK), at least 60 hours prior to implantation. The dose of LY341495 application was 0.40 µg/day, which was calculated by a NTS microinjection study of this antagonist: 15 min drug effect with approximately 2 ng (6 pmol) unilateral injection [23]. Sham surgery with a catheter but without a mini-osmotic pump was performed on SHRs as a sham control.

### 2.3. Measurement of BP and HR

The BP and HR of conscious animals were measured with the tail-cuff method (BP-98AL, Softron Co., Ltd., Tokyo, Japan) 3 days before surgery and at least once a week after surgery for an initial time-course experiment, until 15 weeks of age. Details of the operating procedures have been described in our previous study [24].

### 2.4. RNA Isolation and Quantitative Real-Time Polymerase Chain Reaction (PCR)

For PCR and catecholamine experiments, other SHRs were decapitated after 6 weeks of antagonist treatment under deep isoflurane anesthesia, and blood samples were collected from the left superior vena cava for the measurement of catecholamine concentration, described later. Whole medulla oblongata specimens were dissected, and total RNA was isolated using TRIzol reagent (Gibco-BRL, Grand Island, NY, USA). First-strand cDNA was synthesized using SuperScript IV VILO Master Mix with ezDNase Enzyme (Invitrogen, Carlsbad, CA, USA). With cDNA as a template, real-time PCR was performed with THUNDERBIRD SYBR qPCR Mix (Toyobo, Osaka, Japan) and LightCycler (Roche, Mannheim, Germany). The following primers for real-time PCR were designed based on published sequences: rat glyceraldehyde 3-phosphate dehydrogenase (GAPDH, an internal control) forward primer, 5′-TCA CCA CCA TGG AGA AGG-3′; reverse primer, 5′-GCT AAG CAG TTG GTG GTG CA-3′; rat mGluR2 forward primer, 5′-CGT GAG TTC TGG GAG GAG AG-3′; reverse primer, 5′- GCG GAC CTC ATC GTC AGT AT-3′; rat mGluR3 forward primer, 5′-GTG GTC TTG GGC TGT TTG TT-3′; reverse primer, 5′-GCA GCA TGT GAG CAC TTT GT-3′.

### 2.5. Echocardiographic and Renal Ultrasonographic Measurements

In a separate experiment, echocardiography tests and renal ultrasonography tests were performed in 18-week-old SHRs treated with LY341495 and normotensive control rats, WKY, under 2% isoflurane anesthesia in air with a flow rate of 1 L/min, by using a preclinical imaging system (Vevo 3100, FUJIFILM VisualSonics, Toronto, ON, Canada) and a linear array transducer (MS-550S, FUJIFILM VisualSonics, Toronto, ON, Canada). Echocardiography recordings were made with the preclinical imaging system [25]. Image analysis was performed for left ventricular short-axis, left ventricular inflow waveform and mitral valve septal tissue waveform. Parameters of cardiac function, such as HR, ejection fraction (EF) and cardiac output (CO) were calculated using an analysis software (Vevo LAB, FUJIFILM VisualSonics, Toronto, ON, Canada). Parameters of both sides of the renal artery, such as resistive index (RI) and pulsatility index (PI), that indicate the severity of renal dysfunction were also obtained. An RI higher than 0.75 or a PI higher than 1.55 implies chronic renal failure [26].

### 2.6. Measurement of Catecholamine Concentration

The catecholamine concentration in blood serum was assessed with an ELISA kit (Cat Combi ELISA RUO EIA-4309R, DRG Instruments GmbH, Marburg, Germany) in accordance with the manufacturer’s instructions. Data were compared with reference values obtained from SHRs of a similar age.

### 2.7. Implantation of Telemetry Device for Electrocardiography Recording

After the end of LY341495 treatment, or sham treatment in a separate set of animals, an ECG telemetry device (ATE-01S, Softron Co., Ltd., Tokyo, Japan) was implanted in the backs of several SHRs under isoflurane anesthesia. Paired wire electrodes of the transmitter were subcutaneously placed in the dorsal and ventral thorax to record the apex-base lead ECG. Recordings were performed at 14 weeks of age with a signal receiving board (ATR-1001, Softron Co., Ltd., Tokyo, Japan) that was placed underneath each cage in a temperature- and lighting-controlled chamber (24 °C, 0800–2000 h, MIR-554, Panasonic, Japan). An ECG processor system (Softron Co., Ltd., Tokyo, Japan) was used to continuously record ECG signals.

### 2.8. HRV Analysis

Time- and frequency-domain methods were used to assess autonomic nervous activity. The time-domain analysis was based on R-R intervals for calculating standard deviation (SD) and coefficient of variation (CV), which are regarded as the indices of parasympathetic activity. In the frequency-domain method, power spectral analysis of HRV was performed as previously described [27,28]. Power spectral components were primarily classified into low (LF; 0.04 to 1.0 Hz) and high (HF; 1.0 to 3.0 Hz) frequency ranges as different elements of autonomic nervous activities. The normalized power spectral components of low frequency and high frequency (LFnu and HFnu, respectively) were also calculated to diminish the influence of extremely low frequencies, and highlight the interaction between sympathetic and parasympathetic nerves. LF is affected by both sympathetic and parasympathetic nervous activities, HF is as an index of the parasympathetic nervous activity, and the ratio of LF to HF (LF/HF) is an index of the balance of the autonomic nervous system.

### 2.9. Assessment of Baroreflex Sensitivity

After LY341495 treatment was completed at 12 weeks of age, the implants were removed, and some animals were subjected to invasive catheterization. Under urethane anesthesia (1.5 g/kg, i.p.), i.e., not showing pain reflexes induced by pinching their paws, the animals were placed in the supine position, and a polyethylene catheter was inserted into the left femoral artery to measure arterial blood pressure. The left femoral vein was also cannulated for intravenous administration of pharmacological agents.

Arterial BP was recorded from the catheterized femoral artery with a catheter-transducer system (Nihon Kohden, Tokyo, Japan), which was connected to a computer acquisition system (Softron Co., Ltd., Tokyo, Japan) during the entire measurement. The mean arterial pressure (MAP) was calculated from measured systolic and diastolic BP. Lead II electrocardiographic recordings were obtained with needle electrodes. After 5 min of baseline control recording, an intravenous injection of phenylephrine (PE, 21 µg/kg) or sodium nitroprusside (SNP, 50 µg/kg) was performed through the venous catheter [29,30]. Recordings were continued for 15 min. Baroreflex sensitivity was evaluated with changes in MAP (ΔMAP) and corresponding HR changes (ΔHR) at the peak responses to PE or SNP application. Peak responses were calculated, compared to the 5 min baseline data. As PE increases MAP, and SNP decreases MAP, the HR responses are defined as reflex bradycardia and reflex tachycardia, respectively [29].

### 2.10. Statistical Analysis

All data are expressed as mean ± SEM unless otherwise stated. Changes in BP and HR between antagonist treatment and sham operation in SHRs were evaluated with a two-way repeated measures ANOVA followed by Tukey’s HSD post hoc test when available. Other types of data, such as expression levels of mRNA and HRV, were evaluated with an unpaired *t*-test. JMP^®^ 14 (SAS Institute Inc., Cary, NC, USA) was used for all statistical analysis. *p*-values less than 0.05 were considered statistically significant.

## 3. Results

### 3.1. BP and HR Changes

**Figure 1:**
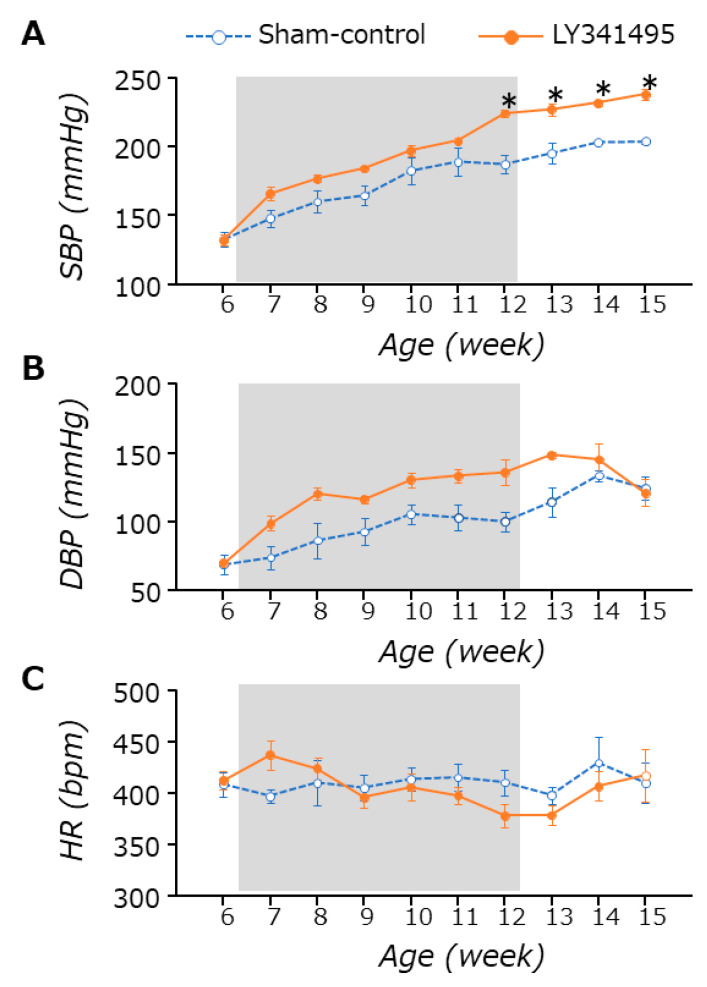
Time-course changes of systolic and diastolic BP (**A**,**B**) and HR (**C**) measured in SHRs. LY341495 treatment (shaded area) was given between the age of 6 and 12 weeks. The treatment increased BP, especially SBP, but did not affect HR. The increased blood pressure was still observed in both groups 3 weeks after the treatment ended. * *p* < 0.05. Statistical evaluations were performed using a two-way repeated measures ANOVA followed by Tukey’s HSD *post hoc* test. Data for each group are the mean ± SEM from five rats.

LY341495 treatments did not affect food intake or body weight in both SHRs and WKYs (data not shown). As already well known, BP, especially systolic BP (SBP), increased with age in SHRs, almost reaching plateau levels after 12 weeks of age (Figure 1). LY341495 (mGluR2/3 antagonist) treatment significantly increased the SBP at 12 weeks of age (sham control vs. antagonist, 187.4 ± 6.3 vs. 224.0 ± 2.8 mmHg) (two-way repeated measures ANOVA: treatment, *p* = 0.005; time, *p* < 0.001; interaction, *p* < 0.001). The difference was still observed at 15 weeks of age, which was 3 weeks after the treatment ended (sham control vs. antagonist, 203.8 ± 1.9 vs. 238.0 ± 4.0 mmHg) (Figure 1A). LY341495 treatment also increased the DBP, compared to that of sham control SHRs during the treatment period (sham control vs. antagonist, 100.0 ± 7.2 vs. 135.6 ± 9.2 mmHg at 12 weeks of age, respectively), but the effects were not much greater than those found in SBPs (two-way repeated measures ANOVA: treatment, *p* = 0.001; time, *p* < 0.001; interaction, *p* = 0.108) (Figure 1B). The antagonist effect was not observed in HR (two-way repeated measures ANOVA: treatment, *p* = 0.732; time, *p* = 0.168; interaction, *p* = 0.134) (Figure 1C). Interestingly, the antagonist effect on SBP was also found in WKYs, the control strain of SHRs, but not on DBP or HR (Supplemental Figure S1). However, as worsening hypertension could be clinically more important, we tried to elucidate the mechanisms of the mGluR2/3 antagonist effect on BP in SHRs in the following experiments.

### 3.2. mGluR2/3 Expression in Medulla Oblongata in SHR

**Figure 2:**
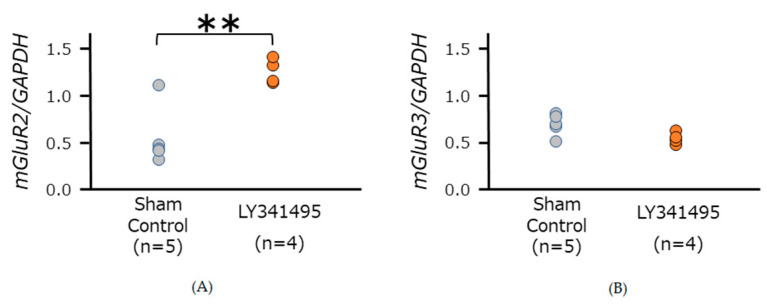
Relative mRNA expression levels of mGluR2 and mGluR3 in the medulla oblongata of 12-week-old sham control and LY341495 treated SHRs. Each mRNA expression level was estimated by real-time PCR, using the GAPDH house-keeping gene expression. Greater expression of mGluR2 mRNA was observed in LY341495 treated SHRs (**A**), but no difference was found in mGluR3 mRNA expression (**B**). ** *p* < 0.01. Statistical evaluations were performed using an unpaired *t*-test. The numbers in parentheses indicate the number of animals.

LY341495 treatment significantly increased mGluR2 expression (unpaired *t*-test, *p* = 0.004), while no significant difference was observed in mGluR3 mRNA expression (unpaired *t*-test, *p* = 0.056) (Figure 2). Before decapitation, the SBP were 211.5 ± 7.7 mmHg in SHRs treated with LY341495 (n = 4), and 186.0 ± 4.8 mmHg in sham control SHRs (n = 5) (unpaired *t*-test, *p* = 0.022).

### 3.3. Echocardiography and Renal Ultrasonography

Table 1 and Supplemental Figure S2 show various parameters of echocardiography and renal ultrasonography of 18-week-old normotensive (Wistar Kyoto) rats and 18-week-old SHRs treated with LY341495 (SBP at the age of 17 weeks, mean ± SD; 113.0 ± 10.0 mmHg and 230.0 ± 4.8 mmHg, respectively). As expected, SHRs with LY341495 treatment showed relatively high HR, stroke volume and cardiac output but were not significantly greater than that in Wistar Kyoto rats. Peak systolic velocity of the renal artery in both sides, a renal parameter, was slower than in normotensive rats. Relatively lower values of both resistive and pulsatility indices of the renal artery were found in SHRs. However, all of the changes in renal parameters did not suggest abnormality, in terms of blood pressure regulation.

### 3.4. Autonomic Nervous System Function during mGluR2/3 Antagonist Treatment in SHRs

**Figure 3:**
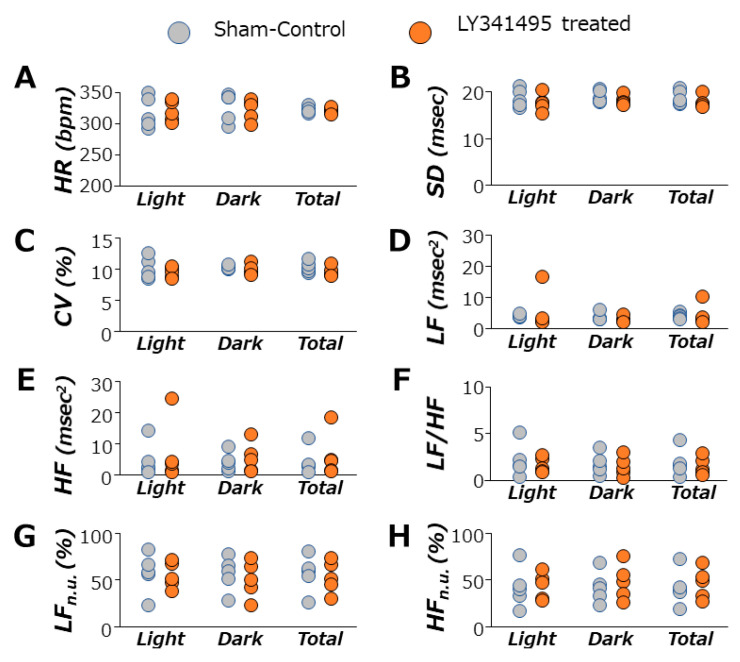
Photoperiod data of autonomic nervous function in 14-week-old SHRs. LY341495 treatment had no significant effect on any parameters of HRV. (**A**), heart rate; (**B**), standard deviation; (**C**), coefficient of variation; (**D**), low-frequency power; (**E**), high-frequency power; (**F**), LF to HF ratio; (**G**), normalized low-frequency power; (**H**), normalized high-frequency power. Data for each group are the mean ± SEM from five rats. Light, 12 h light phase; Dark, 12 h dark phase; Total, 24 h.

Figure 3 shows data for time- and frequency-domain analysis of heart rate variability (HRV) in 14-week-old SHRs during 12 h light phases, 12 h dark phases, and total 24 h phases. mGluR2/3 antagonist treatment did not change the standard deviation of mean heart rate or coefficient of variations, and thus it seemed that there were no changes in HRV time-domain analysis data. Frequency-domain analysis of HRV showed that the antagonist treatment had little effect on LF, HF or LH/HF, suggesting that the autonomic balance was not disturbed. The SBP of the animals used in this HRV study were 220.0 ± 3.0 mmHg in SHRs treated with LY341495 (n = 5), and 200.2 ± 3.1 mmHg in sham control SHRs (n = 5) (unpaired *t*-test, *p* = 0.002).

### 3.5. Catecholamine Blood Test in SHRs Treated with LY341495

Adrenaline and noradrenaline concentrations were treated with LY341495 (mean ± SD; n = 4) 2.48 ± 0.30 ng/mL and 2.64 ± 1.22 ng/mL in SHRs, respectively. These values were quite higher than the reference values of control SHRs (7 to 14 weeks old): 0.018–0.505 ng/mL for adrenaline and 0.120–0.426 ng/mL for noradrenaline [31,32,33].

### 3.6. Effects of Dorsal Hindbrain Treatment with mGluR2/3 Antagonist on Baroreflex Function

**Figure 4:**
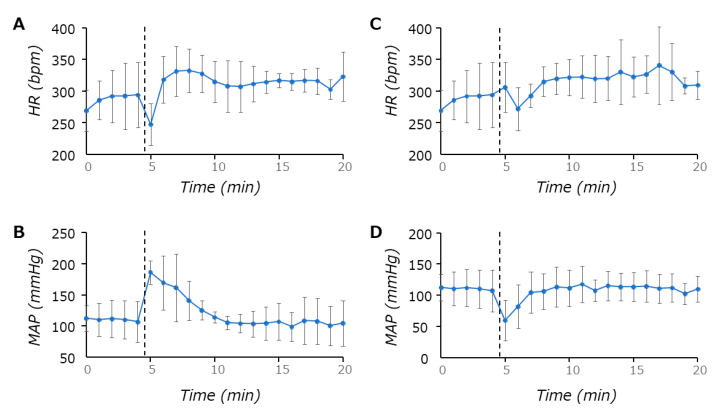
Baroreflex sensitivity in SHRs treated with LY341495. The HR and mean arterial pressure (MAP) responses to intravenous injection of phenylephrine (21 μg/kg) (**A**,**B**), or sodium nitroprusside (50 μg/kg) (**C**,**D**) were plotted every minute. The vertical dashed lines indicate the onset time of phenylephrine or sodium nitroprusside injection. Note that reflex tachycardia was not significantly evident, even though MAP greatly dropped. The data are mean ± SEM from four rats.

Before performing the baroreflex function experiment, the SBP of conscious SHRs treated with LY341495 were measured with the tail-cuff method, and hypertension was confirmed by their values (mean ± SD; 214.0 ± 6.1 mmHg). Phenylephrine increased BP, followed by a transient decrease in HR (reflex bradycardia: (mean ± SD; n = 4) −0.63 ± 0.64 [bpm/mmHg]), while sodium nitro nitroprusside decreased BP followed by a slight increase in HR (reflex tachycardia: (mean ± SD; n = 4) −0.70 ± 1.01 [bpm/mmHg]) (Figure 4). Compared to previously published baroreflex function data of SHR (−0.54 to −1.4 (bpm/mmHg)) [34,35,36], the blockade of endogenous glutamate on mGluR2/3 did not seem to change baroreflex function in SHRs.

## 4. Discussion

Many studies have shown that a microinjection of mGluR modulators into the NTS can change blood pressure [16,17,18]. Although this method can reveal the detailed neuronal mechanisms of blood pressure regulation, its clinical application is quite unrealistic. In this study, we used dorsal hindbrain treatment using an osmotic pump device for a 6-week chronic application, which did not cause any critical changes in food intake or body weight. Although this application method cannot target the NTS selectively, pharmacological effects most likely arise from the NTS, where the blood brain barrier is known to be incomplete [37]. In the current study, mGluR2/3 antagonist treatment in the dorsal hindbrain could not suppress hypertension development in SHRs, but rather exacerbated it by more than 30 mmHg. Interestingly, the exacerbation effects were still observed at 15 weeks of age, which was 3 weeks after the treatment ended, suggesting that the set point of blood pressure was somehow “memorized” during the treatment period and was maintained thereafter. Meanwhile, as the antagonist treatment inhibits endogenous glutamate signaling on mGluR2/3, the activity of this signaling pathway can be crucial for blood pressure regulation, and may become a pharmaceutical treatment target for patients with hypertension.

Earlier NTS microinjection studies mentioned above have some discrepancies. For instance, some studies showed that both group II mGluR agonists and antagonists had similar effects on blood pressure [16,17,18], while others showed that both group I and group II agonists had hypotensive effects, even though these agonists have reciprocal effects on membrane potentials of NTS neurons [11,15]. Thus, we initially suspected that mGluR2/3 antagonists could have a therapeutic potential for hypertension treatment. Although this idea was negated, our results were consistent with our previous study, which showed mGluR2/3 “agonists” could suppress hypertension development in young SHRs [22]. Thus, mGluR2/3 could be important for hypertension development in SHRs and these receptors, and/or neurons expressing these receptors, may have memory functions in blood pressure regulation. This memory function may arise from tonically active neurons in the commissural portion of the NTS which have a crucial role in hypertension [38], however this remains to be elucidated.

In this study, we found that mRNA expression of mGluR2 but not mGluR3 was enhanced by LY341495 treatment in SHRs. This may suggest that even though mGluR2 and mGluR3 are grouped into the same category as “group II mGluRs”, these two receptors could have different functions, requiring selective pharmacological agents to distinguish their individual receptor properties. Furthermore, our results may suggest that mGluR2 but not mGluR3 could be somehow responsible for the exacerbation of hypertension. As mGluR2 signaling was blocked during hypertension development, which was happening from 6 weeks to 12 weeks of age in SHRs, it is possible that some kind of compensating mechanism is recruited to increase the mGluR2 expression in order to receive the signaling.

Exacerbated hypertension by mGluR2/3 antagonist treatment corresponded to higher levels of blood catecholamine, especially adrenaline. In the frequency-domain analysis of HRV, HF is greatly affected by the activity of cardiac parasympathetic nerves [28], while LF is influenced by both vagal and sympathetic nervous activities [39], and therefore LF/HF represents autonomic balance. However, the antagonist treatment did not seem to change the autonomic balance in this study. Thus, the loss of group II mGluR signaling increased the blood catecholamine as well as blood pressure, without significant changes in the autonomic nervous activity or sympathetic–parasympathetic balance.

On the other hand, as we did not see any notable changes in baroreflex function in SHRs, a mechanism(s) for exacerbated hypertension may not be involved in baroreflex pathways. In fact, group II mGluRs have inhibitory effects on glutamatergic signal transmission, which also takes place at the baroreflex arc in the dorsal medulla oblongata [14,15]. Thus, blocking mGluR2/3 signaling would enhance baroreflex and possibly decrease sympathetic outflow, resulting in a decrease in blood pressure. Therefore, neurons which have glutamatergic projections onto RVLM neurons, such as NTS neurons from carotid chemoreceptors [40,41,42,43,44], may be targeted by the antagonist to enhance their neuronal excitability, resulting in the activation of sympathetic outflow. However, as the application area of the antagonist treatment in this study was not strictly limited to the dorsal medulla oblongata, it is not excluded that the antagonist may directly affect the RVLM neurons in the ventral side of medulla oblongata to block the mGluR2/3 mediated inhibitory effects. Another possibility is that a mechanism in which synaptic transmission from GABAergic interneurons in the NTS, was not suppressed by presynaptic group II mGluRs [45]. If these inhibitory interneurons were more active, baroreflex signaling may have been diluted, eventually increasing sympathetic outflow. However, this might not be the case, as the pharmacodynamic effects of the antagonist may be similar for both types of synapses, as the effects of half maximal concentrations of mGluR2/3 agonists were not much different between the presynaptic sites of glutamatergic and GABAergic synapses [14,45].

According to Viard and Sapru (2002) [18], a microinjection of (2R,4R) 4-aminopyrrolidine-2,4-dicarboxylate ((2R,4R)-APDC), an mGluR2/3 agonist, into the NTS decreased the blood pressure transiently (a minute or so) and the effects were blocked by LY341495. In addition, the LY341495 microinjection itself seemed to cause a long-lasting increase in blood pressure, following a transient drop of blood pressure. This phenomenon agrees with our current results, suggesting the pharmacological targets could be in the NTS. Adjacent to the NTS, the area postrema can also be involved in blood pressure regulation. However, as the removal of the area postrema could not affect blood pressure or its set point in adult SHRs, the group II mGluR mechanism found in this study may not be involved in this area [34].

It is not surprising that echocardiographic analysis showed greater cardiac output in SHRs than normotensive rats of a similar age/weight [46] because of higher HR and possibly greater stroke volume [47]. Ultrasonography of the kidney has demonstrated that SHRs treated with LY341495 showed lower peak systolic velocity of the renal artery, compared to normotensive rats. Pulsatility index and resistive index also showed lower values in SHRs. Taken together, these parameters did not indicate the abnormality of cardiac and renal circulation in SHRs treated with LY341495, even though they showed exacerbated hypertension. However, animals used in the current study were not aged, and it would usually take longer until kidney or cardiac malfunction is detectable, unless some sort of intervention is applied, such as diet modification.

Adrenaline and noradrenaline are generally known to be released into the blood stream from chromaffin cells in the adrenal medulla, and these catecholamines may act on their corresponding receptors in various tissues/organs. Interestingly, several neurons in the hindbrain dorsal vagal complex, including the NTS, are immunoreactive to dopamine-β-hydroxylase and phenylethanolamine N-methyltransferase, which are enzymes that convert dopamine to norepinephrine and synthesize adrenaline from noradrenaline, respectively [48]. If these neurons were activated by LY341495 treatment in SHRs, catecholamine production may be upregulated, not only in sympathetic nervous systems but also in other systems. However, this should be further investigated in future studies.

In conclusion, group II mGluR signaling in the dorsal medulla oblongata is necessary to prevent hypertension from worsening in SHRs, which could be mediated by sympathoexcitation, based on data on catecholamine. The mechanism of exacerbated hypertension seemed to be continual and thus certain memory functions may be involved. Therefore, this mechanism(s) can be a therapeutic target for long-lasting blood pressure management in a normal range, and further studies need to be conducted in the future.

## Figures and Tables

**Table 1:** Parameters of Echocardiography and Renal Ultrasonography.

Parameters (Unit)	Wistar Kyoto Rats (n = 5)	SHRs (with LY341495) (n = 5)	*p*-Value (*t*-Test)
*Cardiac*			
Heart Rate (BPM)	329.5 ± 14.0	343.4 ± 15.2	0.520
Stroke Volume (µL)	213.4 ± 9.5	241.0 ± 20.9	0.264
Ejection Fraction (%)	69.1 ± 2.8	61.6 ± 3.6	0.138
Fractional Shortening (%)	40.3 ± 2.2	34.8 ± 2.7	0.147
Cardiac Output (mL/min)	70.3 ± 4.1	81.8 ± 5.1	0.117
*Renal*			
LRA PSV (mm/s)	780.6 ± 86.3	440.8 ± 100.6	0.033 *
RRA PSV (mm/s)	786.3 ± 65.5	544.2 ± 74.0	0.040 *
LRA LDV (mm/s)	237.4 ± 34.0	207.9 ± 46.1	0.621
RRA LDV (mm/s)	231.7 ± 41.8	215.4 ± 25.6	0.748
LRA PI	1.304 ± 0.276	0.746 ± 0.042	0.081
RRA PI	1.377 ± 0.200	0.905 ± 0.071	0.057
LRA RI	0.673 ± 0.060	0.526 ± 0.023	0.052
RRA RI	0.701 ± 0.048	0.596 ± 0.021	0.080

LRA, left renal artery; RRA, right renal artery. PSV, peak systolic velocity; LDV, lowest diastolic velocity. PI, pulsatility index; RI, resistive index. * *p* < 0.05.

## Data Availability

Not applicable.

## Supplementary Materials

The following are available online at https://www.mdpi.com/article/10.3390/life11070720/s1, Figure S1: Time course changes of systolic and diastolic blood pressure (BP) and heart rate (HR) measured in Wistar Kyoto rats. LY341495 treatment (shaded area) was given during the period from the age of 6 to 12 week-old. The treatment slightly increased SBP from the middle of the antagonist treatment (two-way repeated measures ANOVA: treatment, *p* < 0.001; time, *p* < 0.001; interaction, *p* < 0.001), suggesting loss of group II mGluR signaling has a similar effect on blood pressure regulation regardless of the normotensive/hypertensive condition. (DBP: treatment, *p* = 0.001; time, *p* = 0.023; interaction, *p* = 0.002) (HR: treatment, *p* = 0.221; time, *p* = 0.002; interaction, *p* = 0.052) * *p* < 0.05; statistical evaluations were performed using two-way repeated measures ANOVA followed by Tukey’s HSD post hoc test. Data for each group are the mean ± SEM from five rats; Figure S2: Recording images from echocardiography and renal ultrasonography. The left panel shows a color doppler view of mitral flow and its velocity (A) and the right panel shows a color doppler image of renal artery and its systolic velocity (B).

## Abbreviations

AMPA: α-amino-3-hydroxy-5-methyl-4-isoxazolepropionic acid; ANOVA, analysis of variance; BP, blood pressure; CO, cardiac output; CVLM, caudal ventrolateral medulla; DBP, diastolic blood pressure; ECG, electrocardiography; EF, ejection fraction; GABA, γ-aminobutyric acid; HF, high frequency; HR, heart rate; HRV, heart rate variability; LF, low frequency; MAP, mean arterial pressure; mGluR, metabotropic glutamate receptor; NMDA, N-Methyl-d-aspartic acid; NTS, nucleus tractus solitarius; PE, phenylephrine; PI, pulsatility index; RA, renal artery; RI, resistive index; RM, repeated measurement; RVLM, rostral ventrolateral medulla; SBP, systolic blood pressure; SHRs, spontaneously hypertensive rats; SNP, sodium nitroprusside; WKYs, Wistar Kyoto rats.
