# Supplementary material for: Loss of Group II Metabotropic Glutamate Receptor Signaling Exacerbates Hypertension in Spontaneously Hypertensive Rats
Source: Life (Basel). 2021 Jul 20;11(7):720. doi: 10.3390/life11070720 (PMC8307370; doi:10.3390/life11070720)
Supplement: Supplementary file 1 [file life-11-00720-s001.zip › life-1298606-supplementary0721-reply.pdf]

life

MDPI

Article

Loss of Group II Metabotropic Glutamate Receptor Signaling Exacerbates Hypertension in Spontaneously Hypertensive Rats

Julia Chu-Ning Hsu [1], Shin-ichi Sekizawa [1*], Ryota Tochinai [1] and Masayoshi Kuwahara [1*]

[1] Department of Veterinary Pathophysiology and Animal Health, Graduate School of Agricultural and Sciences, The University of Tokyo, Tokyo 113-8654, Japan
* Correspondence: akuwam@mail.ecc.u-tokyo.ac.jp (M.K.), a-ssekiz@mail.ecc.u-tokyo.ac.jp (S.S.)

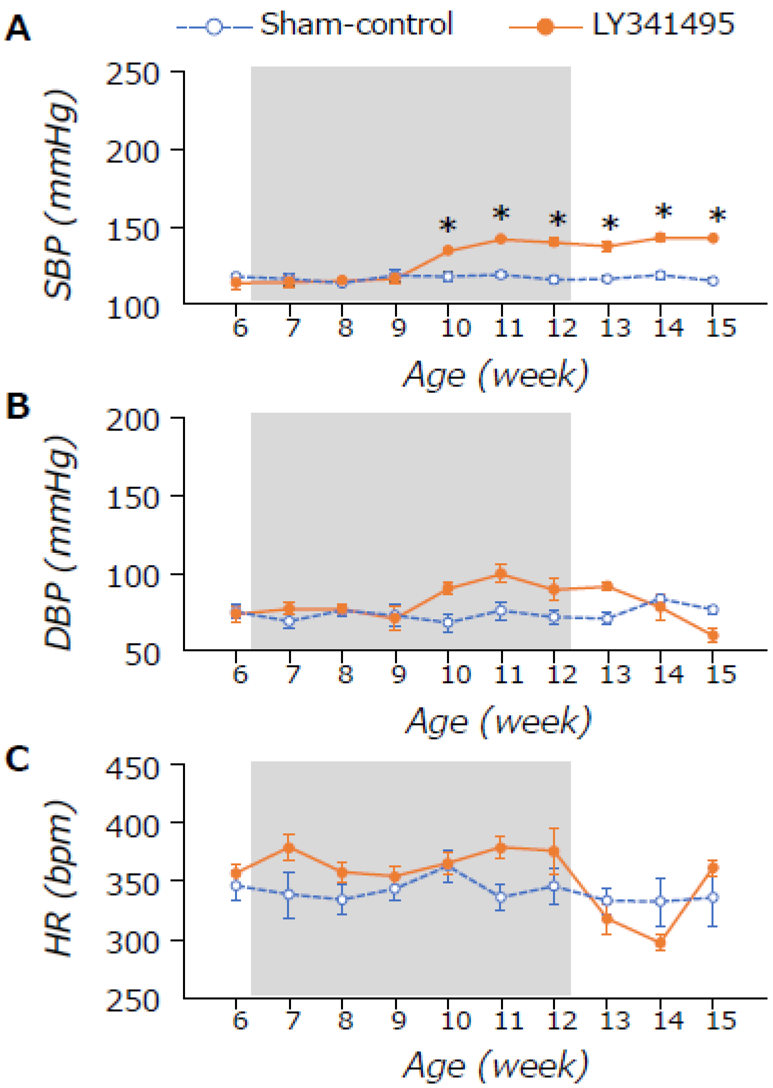

Figure S1. Time course changes of SBP (A), DBP (B) and HR (C) measured in Wistar Kyoto rats. LY341495 treatment (shaded area) was given between the age of 6 and 12 weeks. The treatment slightly increased SBP from the middle of the antagonist treatment (two-way repeated measures ANOVA: treatment, $p < 0.001$; time, $p < 0.001$; interaction, $p < 0.001$), suggesting loss of group II mGluR signaling has a similar effect on blood pressure regulation regardless of the normotensive/hypertensive condition. (DBP: treatment, $p = 0.001$; time, $p = 0.023$; interaction, $p = 0.002$) (HR: treatment, $p = 0.221$; time, $p = 0.002$; interaction, $p = 0.052$) *$P < 0.05$; statistical evaluations were performed using two-way repeated measures ANOVA followed by Tukey's HSD *post hoc* test. Data for each group are the mean ± SEM from five rats.

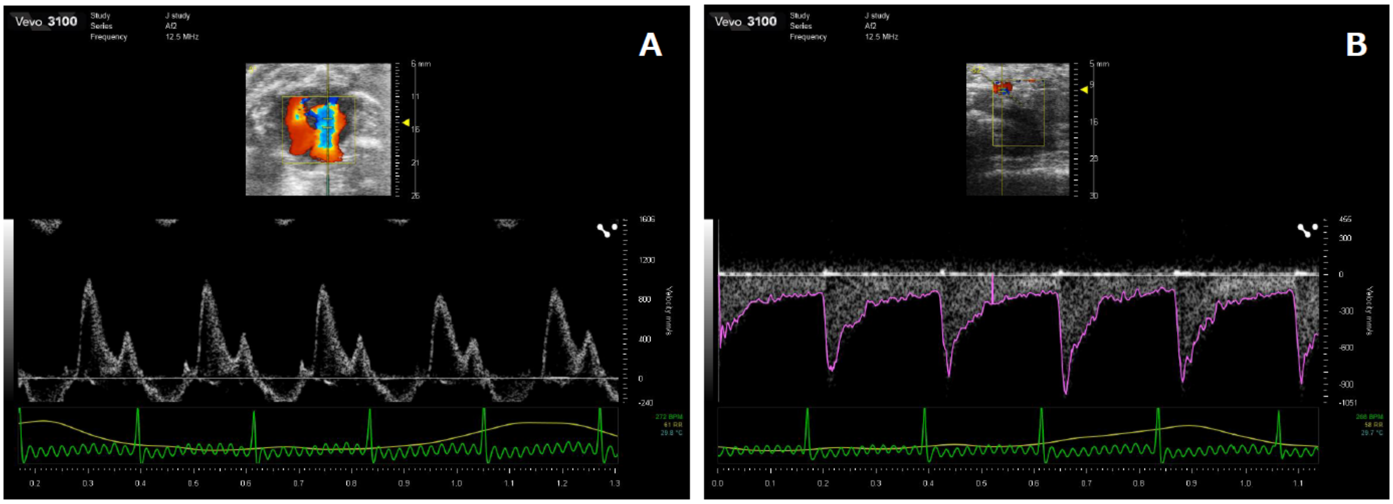

Figure S2. Recording images from echocardiography and renal ultrasonography. The left panel shows a color doppler view of mitral flow and its velocity (**A**) and the right panel shows a color doppler image of renal artery and its systolic velocity (**B**).
